# Tracheomalacia in Adults: An Uncommon Cause of Dyspnea

**DOI:** 10.7759/cureus.49190

**Published:** 2023-11-21

**Authors:** Yusur Alsalihi, Natalie M Yousef, Sundeep Grewal, Benjamin Teitelbaum

**Affiliations:** 1 Clinical Education Department, California Health Sciences University College of Osteopathic Medicine, Clovis, USA; 2 Clinical Education Department, Internal Medicine, California Health Sciences University College of Osteopathic Medicine, Clovis, USA; 3 Surgery Department, Clovis Community, Clovis, USA

**Keywords:** cartilaginous disorders, general internal medicine, dyspnea of unknown origin, tracheomalacia, otolaryngology, ear nose throat (ent)

## Abstract

Tracheomalacia (TM), the most common congenital tracheal defect, is due to compromised cartilage integrity, manifesting in the narrowing of expiratory airways and various respiratory symptoms. While TM is common in infants and toddlers, it is rarely found in adults, often due to acquired injuries or chronic lung diseases. We present a unique case of a 67-year-old man with persistent dyspnea and hoarseness for two years. Despite a history of smoking, he had no signs of pulmonary disease and had a consistently high oxygen saturation during episodes of dyspnea. His dyspnea was of unknown etiology until a diagnostic bronchoscopy revealed tracheal stenosis and flaccidity of cartilaginous structures, with pronounced collapse during expiration. This atypical presentation highlights the complexity of TM in adults. It underscores the importance of considering it as a differential diagnosis, particularly in male smokers with gradual, persistent dyspnea and a minimal history of pulmonary disease.

## Introduction

The normal intrathoracic trachea is compliant, dilating with inspiration and narrowing with expiration due to the difference between intrathoracic and intraluminal pressures [1-3]. However, in tracheomalacia (TM), the tracheal cartilage is compromised, leading to loss of structural integrity and an inability to prevent airway collapse due to increased intrathoracic pressure during exhalation. Most cases of TM are expiratory, indicating excessive tracheal narrowing when intrathoracic pressure is substantially greater than intraluminal pressure such as during forced expiration, cough, or the Valsalva maneuver [4]. Furthermore, various respiratory symptoms, such as chronic cough, wheezing, shortness of breath (dyspnea), noisy breathing (stridor), and recurrent respiratory infections, are common.

Although TM occurs in 1 in 2,100 infants and toddlers [1,5], such a presentation is increasingly rare in the adult population that has fully developed tracheal cartilages, so much so that specific incidence rates in adults (without underlying conditions or predisposing factors) have not been widely reported in the medical literature. TM in the adult population is typically due to an acquired injury from previous surgery involving the airway, intubation, or chronic lung disease. Additionally, chronic compression due to goiter or tumors and masses, which occur most commonly in the middle-aged and elderly, may predispose to the development of TM in adults. The most affected adult demographic with TM is men >40 years of age [6].

This article was previously presented as a poster at the American College of Osteopathic Internists conference in Tampa, Florida, in October 2023.

## Case presentation

We present a case of a 67-year-old Caucasian man who presented to the outpatient ENT clinic with a primary complaint of dyspnea for two years. The patient also complained of hoarseness but felt no changes in the strength of his voice. He was afebrile and stated that he has been healthy for the past two years with no significant illness to report. Furthermore, the onset of dyspnea has been gradual, with no pulmonary or constitutional symptoms. He noticed that shortness of breath occurs randomly without direct association with exercise or exertion. His medical history was negative for pulmonary disease and seasonal allergies.

After ruling out cardiac and pulmonary causes, the patient was referred to an otolaryngologist. On physical examination, the patient was found to have an expiratory stridor. His vitals were within the normal range, and his oxygen saturation was 98%, with no use of accessory muscles or significant respiratory effort. Social history was positive for tobacco use of one pack/week for 30 years, and no significant use of alcohol or drugs was reported. Under suspicion of growth along the oropharyngeal pathway, in-office laryngoscopy was performed. The scope did not show masses or abnormal structural findings.

**Figure 1:**
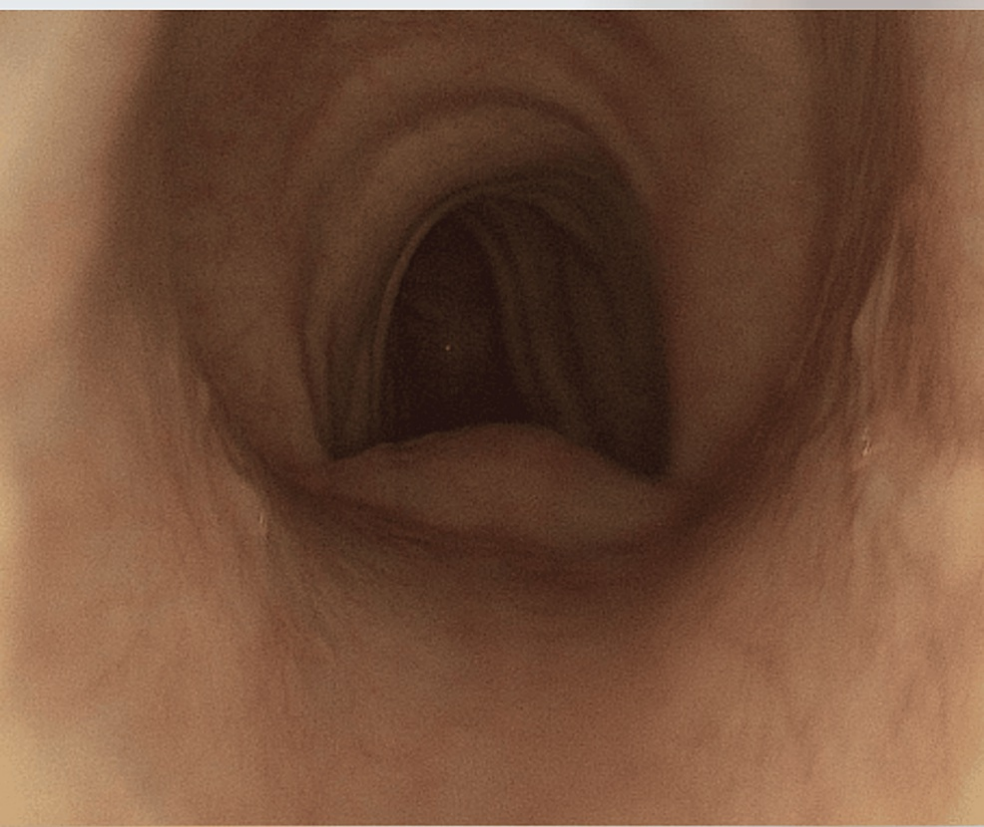
Patent tracheal expansion during the inspiratory phase

**Figure 2:**
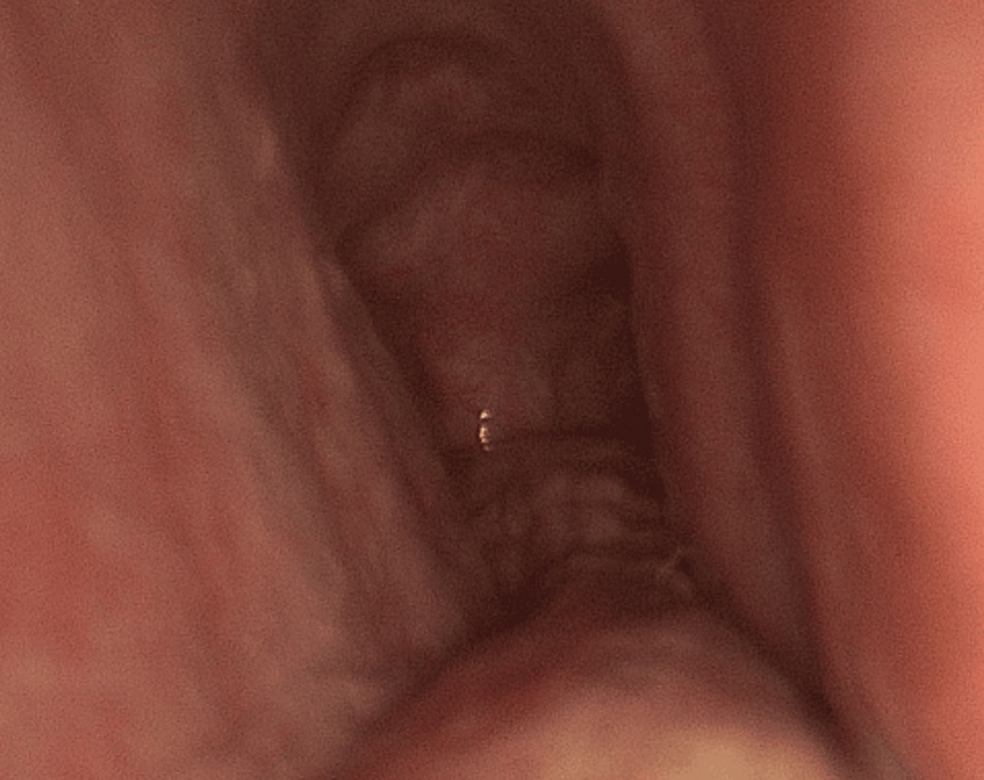
Lateral wall trapezoidal collapse during the expiratory phase

Speech therapy and voice rest were recommended, and the patient was referred for a thorax CT scan that showed transverse tracheal narrowing at level T3 and tracheal stenosis. Under suspicion of compressive structure, the patient underwent a diagnostic endoscopy and bronchoscopy. No masses or growths were observed but rather, an overall flaccidity of cartilaginous structures within the trachea. The flaccidity was more pronounced distal to the carina. The cartilaginous structure was observed to be open during the inspiratory phase (Figure 1) but had a collapse of the lateral wall during the expiratory phase (Figure 2).

## Discussion

Our case presents atypical symptoms with no identifiable direct cause. In the adult population, TM is most often due to an acquired disease process. Tracheostomy or endotracheal tube intubation is the most common cause of secondary TM [4]. Traumatic tracheal injury that causes cartilage loss, including external trauma and surgery, can also cause TM. Our patient had no pulmonary disease or direct trauma to the chest or surrounding structures. 

Some research suggests that chronic inflammation and irritants, such as cigarette smoke, contribute to the development of TM [4,7]. Weakening of the tracheal wall may be related to recurrent injury from irritants. This association is most closely related to the development of COPD due to smoking and, subsequently, tracheal irritation and collapse. Also, it may be due to irritation that causes excessive coughing or increased gag reflex, increasing intrathoracic pressure, and increasing collapsibility. Although our patient is a smoker, there has been no direct explanation of how smoking causes TM, and he did not have signs of COPD or excessive lung damage. While smoking is a contributing factor, it is rare to see isolated TM due to tobacco use [5].

Studies show that smoking is linked with mucosal and cartilaginous irritation and inflammation [8]. Increased inflammation causes an aberrant activation of the immune system and recruitment of inflammatory biomarkers. Some of these biomarkers are tasked with cleaning up the debris, including healthy and unhealthy cells. The process can lead to synechiae and collagenous band formation. These bands can lead to structural reformation of the known cartilaginous-heavy cartilaginous structure. Interestingly, our patient has not been a life-long smoker, and we suspect that his smoking is a contributing factor to his tracheomalacia diagnosis but not the only factor. We hypothesize that while smoking is the source of the irritation, this patient may be more predisposed to aberrant immune response due to his old age and possibly, a genetic component. While he denied any pathological concerns about his musculoskeletal system and joint health, we recognize that such an unusual response may have a multi-factorial origin.

Most interestingly, while our patient felt short of breath, his oxygen saturation remained well above 92% for the duration of the history intake. Furthermore, even when conversing, his oxygenation levels did not drop. It is important to note that the patient's trachea does not collapse on breathing in, but rather on breathing out, indicating that appropriate tidal volume is reaching the lungs, but the expiratory phase is difficult. Akin to breathing out of a straw, we hypothesize that the presumed reduction in expiratory volume due to tracheal collapse leads to increased carbon dioxide within the lungs, increasing respiratory drive in the brain. The increased respiratory drive within the brain and the increased carbon dioxide concentration within the lungs give a sensation of shortness of breath.

Another unique feature of his dyspnea was the constant nature of his symptoms. Typically, pulmonary or cardiac dyspnea worsens with exertion and is relieved by rest, unless in advanced disease processes. This patient felt short of breath even at rest and felt his dyspnea was constant. The stable and gradual nature of this presentation led him to defer going to the doctor for two years. Typically, the presentation of TM in adults requires intubation until a source is identified and corrected or until a tracheoplasty is performed.

Management of this case included smoking cessation and a trial of respiratory exercises. We also recommended humidified air due to some studies that show that it leads to a reduction in the inflammatory response. Currently, the patient is under observation and continued management if his symptoms worsen or improve [9]. 

## Conclusions

In conclusion, our case presents an unusual constellation of symptoms with no apparent cause. Tracheomalacia (TM) in adults is typically acquired, often resulting from tracheostomy or intubation. However, our patient did not have a history of such incidents. Chronic inflammation and irritants, such as cigarette smoke, may contribute to TM, but the patient showed no signs of related conditions. In particular, his oxygen saturation remained above 92% during dyspneic episodes, and his symptoms were constant, unlike typical pulmonary or cardiac-related dyspnea. Clinicians should note the nature of dyspnea, constant versus exerted, and keep TM in their differential diagnosis, especially in men with a positive history of smoking, insignificant pulmonary disease, and gradual, constant symptomatic dyspnea. Careful evaluation and follow-up are necessary to determine the best course of treatment for this rare presentation.
